# A ferroptosis-related gene signature and immune infiltration patterns predict the overall survival in acute myeloid leukemia patients

**DOI:** 10.3389/fmolb.2022.959738

**Published:** 2022-08-15

**Authors:** Zhao Yin, Fang Li, Qinjun Zhou, Jianfang Zhu, Zhi Liu, Jing Huang, Huijuan Shen, Ruiming Ou, Yangmin Zhu, Qing Zhang, Shuang Liu

**Affiliations:** Department of Hematology, Guangdong Second Provincial General Hospital, Guangzhou, China

**Keywords:** ferroptosis, AML, ARNTL, immune cell infiltration, overall survival (OS)

## Abstract

Targeted therapy for acute myeloid leukemia (AML) is an effective strategy, but currently, there are very limited therapeutic targets for AML treatment. Ferroptosis is strongly related to drug resistance and carcinogenesis. However, there are few reports about ferroptosis in AML. This article explores the relationship between ferroptosis-related gene (FRG) expression and prognosis in AML patients from the FerrDb and the Cancer Genome Atlas (TCGA) databases. The ferroptosis-related gene ARNTL was observed to have high expression and poor prognosis in AML. Receiver operating characteristic curve (ROC) analysis revealed the predictive accuracy of the signature. The area under the time-dependent ROC curve (AUC) was 0.533 at one year, 0.619 at two years, and 0.622 at three years within the training cohort. Moreover, we found that the ARNTL expression is closely associated with tumor-infiltrating immune cells like the macrophages and NK cells. Inhibiting the ARNTL expression suppressed colony formation and induced ferroptosis in AML cells. Overall, the survival prediction model constructed based on ARNTL accurately predicted the survival in AML patients, which could be a potential candidate for diagnosing and treating AML.

## Introduction

Acute myeloid leukemia (AML) is a complex hematological neoplasm with a poor prognosis (Willier et al., 2021). This disease is the most frequent type of malignant myeloid disorder in adults with an incidence of approximately 2–4/100,000 per year (http://seer.cancer.gov/). Moreover, patients older than 60 years are not able to withstand induction chemotherapy and have an even worse median survival of 5–10 months, and their 5-year overall survival (OS) is as low as 5% (Lai et al., 2019). Therefore, it is necessary to explore more effective AML therapeutic strategies.

Ferroptosis is an iron-dependent cell death type, which accumulates lipid reactive oxygen species (lipid-ROS) (Dixon et al., 2012). Ferroptosis plays an essential role in regulating cancer development and can be used in antitumor treatment and predicting prognosis (Jiang et al., 2021; Zhang et al., 2022). The induction of ferroptosis becomes a candidate strategy for inducing tumor cell death, particularly in treating resistant cancers (Hassannia et al., 2019; Lei et al., 2022). Studies have shown that some genes, such as Aldh3a2 (Yusuf et al., 2020), p53 (Birsen et al., 2021), and GPX1 (Wei et al., 2020), regulated ferroptosis in cancer cells and affected AML prognosis. Combining chemotherapeutics with erastin, the ferroptosis activator, is suggested to enhance drug efficacy in AML (Yu et al., 2015). Therefore, ferroptosis has a critical effect on AML. Numerous studies have indicated that such abnormally expressed proteins can be adopted as biomarkers to predict cancer prognosis (Wang et al., 2021). Nevertheless, the relationship of such FRGs with AML survival is still unknown.

To the best of our knowledge, in this present study, we comprehensively analyzed the ARNTL expression and correlation with the prognosis of AML patients in databases such as the TCGA and Kaplan–Meier plotter. Moreover, we investigated the correlation of ARTNL with tumor-infiltrating immune cells in the different tumor microenvironments. These findings provide a promising prognostic gene for AML that was developed based on ferroptosis-associated differentially expressed genes that could be used for prognosis prediction and selection of patients for immunotherapies.

## Materials and methods

### Data collection

We downloaded clinical and RNA-seq expression profiles of 173 cancer patients and 70 healthy subjects from the TCGA website. Later, the “limma” function of the R package was utilized to normalize RNA-seq expression patterns.

We also obtained FRGs from the FerrDb database (http://www.zhounan.org/ferrdb/) containing the ferroptosis markers and regulators.

The GeneCards database included integrated information regarding the annotated and predicted human genes. In addition, the ferroptosis-related genes can be downloaded from this database.

The cell line mRNA expression matrix of tumors was obtained from the CCLE dataset (https://portals.broadinstitute.org/ccle).

### Establishment and verification of the FRG-based prognosis model

We adopted the “limma” function from the R package for identifying differentially expressed genes (DEGs) in AML samples compared with healthy controls upon the thresholds of log2|fold change (FC)|>1 and false discovery rate (FDR) < 0.05 based on the TCGA cohort.

### Kaplan–Meier (K–M) and ROC analyses on the prognosis model

We conducted the K–M analysis for determining the difference in OS between high- and low-expression groups for evaluating the prognosis prediction accuracy of our constructed FRG model using the log-rank test with R package “survminer” and “survival” functions (Kassambara et al., 2018). In addition, time-dependent ROC (t-ROC) curves were plotted using the “survival” and “timeROC” of the R package for assessing the accuracy of our prognosis model.

### Cell culture and siRNA silencing

We acquired human AML cells (Molm-13) from Keygentec (Jiangsu, China). Moreover, we obtained ARNTL siRNA from Ribio (Guangzhou, China). Later, Molm-13 cells were cultivated within RPMI-1640 (Gibco, Grand Island, NY, United States) containing 10% fetal bovine serum (FBS) under 5% CO_2_ and 37°C temperature conditions. Later, Lipofectamine 2000 (Invitrogen, Carlsbad, CA, United States) was used to transfect siRNAs (target sequence: GTG​GAA​TCC​TGG​GCC​TTC​ATT, 100 nmol/L) in cells.

### Lipid ROS assay using flow cytometer

Lipid ROS levels were determined using BODIPY-C11 dye (Invitrogen, cat# D3861). The intensely fluorescent BODIPY (4,4-difluoro-3a, 4a-diaza-s-indacene) fluorophore is intrinsically lipophilic, unlike most other long wavelength dyes. The binding of BODIPY fatty acids to bovine serum albumin can be monitored by the accompanying fluorescence quenching caused by charge–transfer interactions with aromatic amino acid residues. BODIPY 581/591C 11 can be used to measure antioxidant activity in lipid environments by exploiting its loss of fluorescence upon interaction with peroxyl radicals. In brief, we inoculated cells (2 × 10^4^/well) into the six-well plates, followed by 48 h transfection with siRNAs (100 nmol/L) using the Lipofectamine 2000. Before the end of time, culture media were replaced with 1 ml media containing 5 μM of BODIPY-C11 dye for 60 min. Cells were harvested and washed twice with PBS, followed by resuspending in 500 μL of PBS, and subjected to the flow cytometry analysis to examine the amount of ROS within cells (Yin et al., 2020a).

### Clone-forming assays

Molm-13 cells were transfected with 100 nmol/L siRNAs for 48 h and then collected. The cells (1 × 10^3^/well) were inoculated into a 10-cm dish with soft agar culture for two weeks to conduct a clone-forming assay with the monolayer cultures. Later, clones were fixed, stained, counted, and photographed (Yin et al., 2020b).

### Malondialdehyde (MDA) assay

MDA accounts for the critical factor indicating lipid peroxidation. Therefore, the MDA levels (S0131S, Beyotime, Shanghai, China) were measured and normalized to protein content according to specific protocols in this study. In brief, cells were harvested and cellular extracts were prepared by sonication in the ice-cold buffer. After sonication, lysed cells were centrifuged at 10,000 × *g* for 20 min to remove debris. The supernatant was subjected to the measurement of MDA levels and the protein contents. We used a BCA kit (BL521A, Biosharp, Anhui, China) to quantify protein concentration. MDA levels were then normalized to milligram protein. We used the same procedure to lyse the cells and determine the protein contents in the following assays unless otherwise indicated.

### RNA isolation and real-time PCR (RT-PCR)

We utilized the Trizol reagent (Takara) for extracting total RNA in cells transfected with siARNTL (Liu et al., 2021). Then, we incorporated PrimeScriptTM RT Master Mix (HY-K0510, MCE, United States) to synthesize cDNA from total RNA (1 μg) following the specific protocols. Using the SYBR-Green kit (B21202, Biomake, Shanghai, China), we measured the ferroptosis marker expression using RT-PCR, including ACSL4 and GPX4, and human *β*-actin as the reference. All the primers utilized in RT-PCR were purchased from Sangon Biotech and included.

GPX4-Forward (5’-3’): ATG​GTT​AAC​CTG​GAC​AAG​TAC​C.

ACSL4-Forward (5’-3’):ACC​AGG​GAA​ATC​CTA​AGT​GAA​G.

GPX4-Reverse (5’-3’): GAC​GAG​CTG​AGT​GTA​GTT​TAC​T.

ACLS4-Reverse (5’-3’):GGT​GTT​CTT​TGG​TTT​TAG​TCC​C

β-Actin-Forward (5’-3’):

CCT​TCC​TGG​GCA​TGG​AGT​C

β-Actin-Reverse (5’-3’):

TGA​TCT​TCA​TTG​TGC​TGG​GTG.

### Western blot assay

Molm-13 cells were collected and lysed with RIPA buffer (BL504A, Biosharp, China) for 30 min on ice. Then, the protein (50 μg) from each sample was separated by 10% tris-acrylamide gel electrophoresis and transferred onto the PVDF membrane. After blocking with 5% skim milk for 1 h at room temperature, primary antibodies against GPX4 (DF6701, Affinity, United States), ACSL4 (DF12141, Affinity, United States), and GAPDH (AF7021, Affinity, United States) were used and incubated at 4°C overnight on a rotary shaker. After washing with TBST 5 times, the membrane was probed with goat anti-rabbit IgG highly cross-adsorbed secondary antibody (1:10,000) for 1 h at room temperature. Then, the membrane was washed 3 times with TBST and developed with an ECL reagent.

### Statistical analysis

R version 3.6.1 was used for statistical analyses. One-way ANOVA was used to analyze the FRG expression between cancer and non-cancer samples. The K–M method was used to generate survival curves, while the log-rank test was used to compare differences. *p* < 0.05 indicated statistical significance (two-sided).

## Results

### Identification of ferroptosis-related genes (FRGs) in AML

**FIGURE 1:**
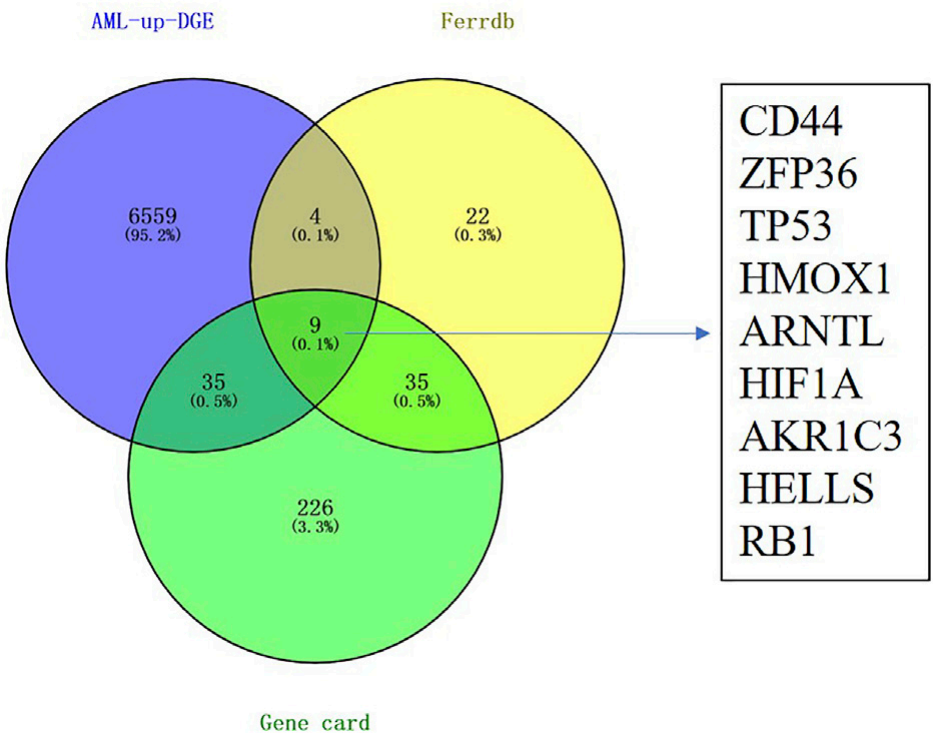
Identification of differentially expressed genes related to ferroptosis in the TCGA LAML cohort. Nine candidate prognostic genes were differentially expressed between tumor and normal.

We extracted the RNA-Seq data of DEGs from AML patients in the TCGA database using the Gene Expression Profiling Interactive Analysis (GEPIA2) (Tang et al., 2019) to reveal the ferroptosis-related genes in AML. We also collected the ferroptosis-related genes from the FerrDb database. We speculated that activating ferroptosis signaling pathways could inhibit AML, so we focus on the ferroptosis suppression gene and its overexpression in AML patients. As shown in Figure 1, we observed that nine genes were involved in the DEGs of AML overexpression, ferroptosis-suppression genes, and ferroptosis-related genes obtained from the GeneCards database (CD44, ZFP36, TP53, HMOX1, ARNTL, HIF1A, AKR1C3, HELLS, and RB1).

### Identification of ferroptosis-related genes associated with OS in AML

**FIGURE 2:**
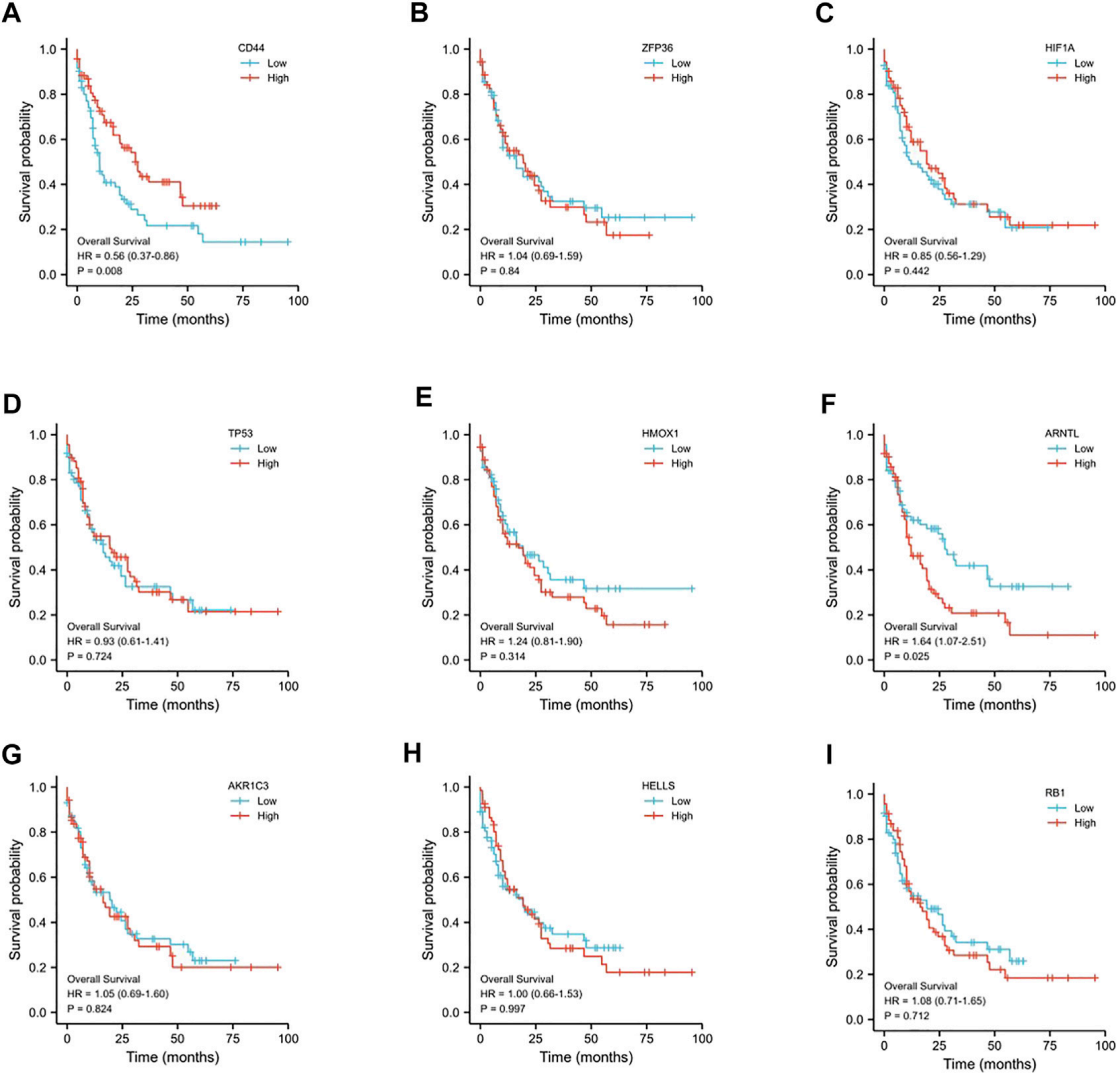
Prognostic value of the 9-ferroptosis-related-gene signature in AML patients. **(A)** Kaplan–Meier curves for the overall survival of high- and low-CD44 expression AML patients; **(B)** Kaplan–Meier curves for the overall survival of high- and low-ZFP36 expression AML patients; **(C)** Kaplan–Meier curves for the overall survival of high- and low-HIF1A expression AML patients; **(D)** Kaplan–Meier curves for the overall survival of high- and low-TP53 expression AML patients; **(E)** Kaplan–Meier curves for the overall survival of high- and low-HMOX1 expression AML patients; **(F)** Kaplan–Meier curves for the overall survival of high- and low-ARNTL expression AML patients; **(G)** Kaplan–Meier curves for the overall survival of high- and low-AKR1C3 expression AML patients; **(H)** Kaplan–Meier curves for the overall survival of high- and low-HELLS expression AML patients; **(I)** Kaplan–Meier curves for the overall survival of high- and low-RB1 expression AML patients. *p* < 0.05 is considered as significant.

**FIGURE 3:**
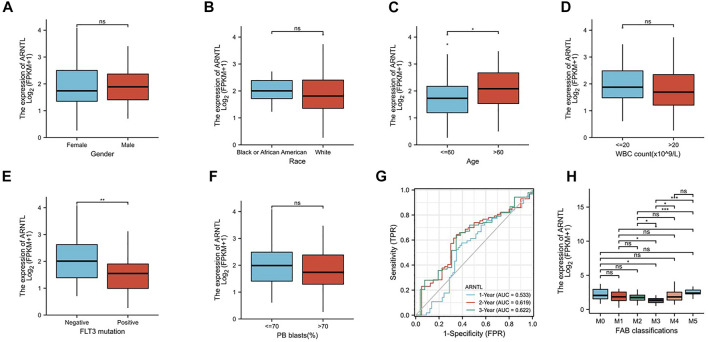
Expression of ARNTL in different stages of AML. **(A)**. Gender, **(B)** race, **(C)** age, **(D)** WBC count, **(E)** FLT3 mutation, **(F)** PB blasts, **(G)** Time-dependent ROC curve of AML patients in the training group and **(H)** FAB classification, *p* < 0.05 is considered as significant. *p < 0.05, **p < 0.01.

We examined OS for AML cases according to FRG levels based on the GEPIA database to determine whether our FRG-based prognosis nomogram was accurate. A threshold of 50% was accepted for dividing low and high values, with *p* < 0.05 indicating statistical significance (Figure 2). ARNTL showed significant relation to OS for AML (*p* = 0.025, Figure 2F), which was identified as a risk factor. Then, we analyzed the expression of ARNTL in pan-cancer, and we found only higher expression of ARNTL in CESC, GBM, HNSC, KIRC, LAML, and THCA (Supplementary Figure S1). According to the timeROC curve analysis (Figure 3G), ARNTL significantly predicted the survival of AML (1-, 2-, and 3-year AUC of 0.533, 0.619, and 0.622, respectively).

### Ferroptosis-related gene signature is related to clinicopathological features

To explore the relationship between the constructed nomogram and clinicopathological features, we analyzed the FRG levels among diverse subgroups that were divided based on gender (Figure 3A), race (Figure 3B), age (Figure 3C), WBC count (Figure 3D), FLT3 mutation (Figure 3E), PB blast (Figure 3F), and FAB classification (Figure 3H). As a result, we found that ARNTL is the lower expression in M3 AML and higher in M5 AML.

When the age of patients was greater than 60, ARNTL had significantly higher expression. However, with the BM blast higher than 20, the ARNTL expression was substantially lower. Interestingly, the FLT3-positive mutation patients have lower ARNTL than those with negative expression.

### Identification of ARNTL associated with clinicopathological features in AML

**FIGURE 4:**
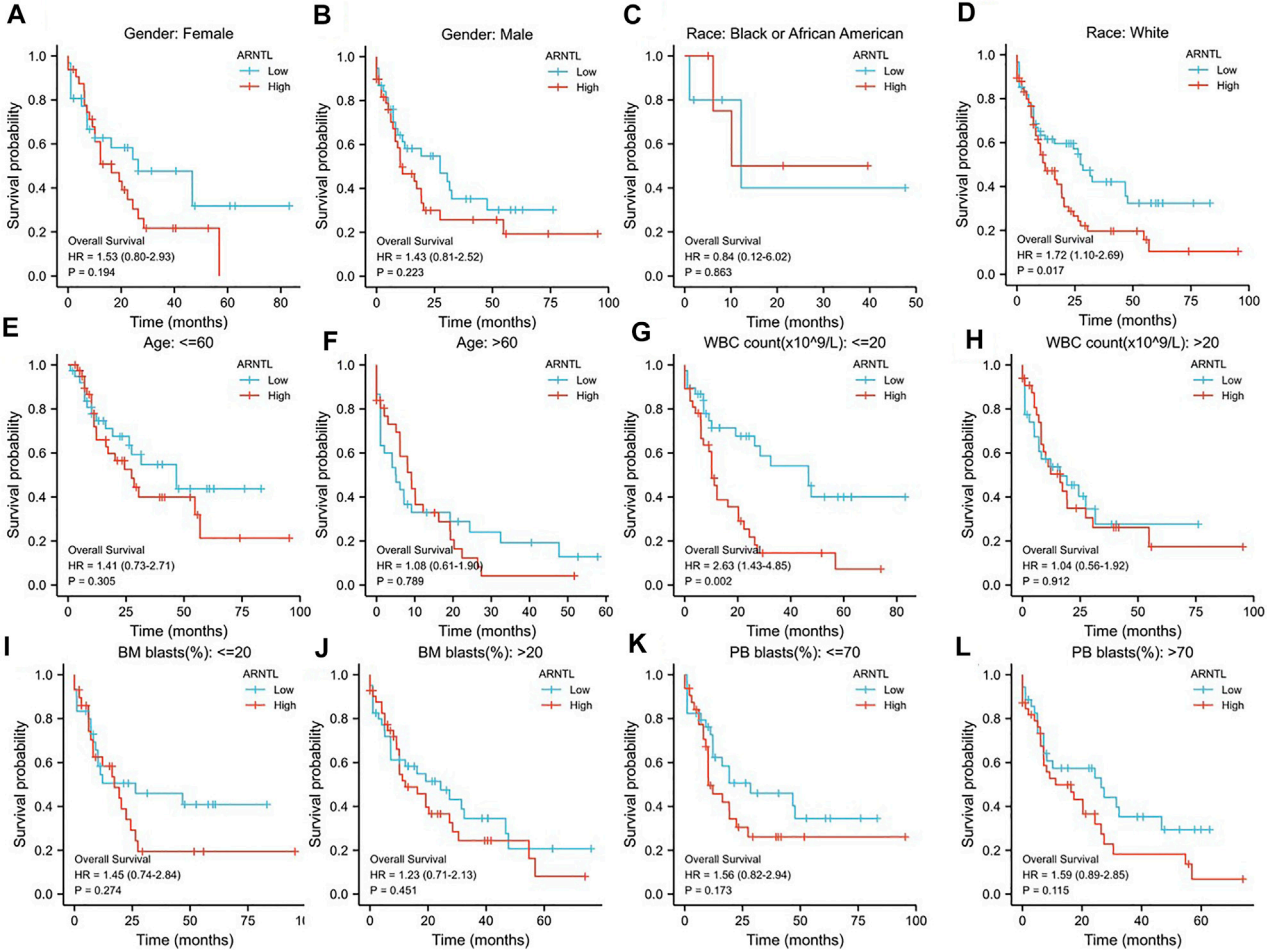
Prognostic value of the ARNTL in different stages of AML. **(A, B)** Gender; **(C, D)** Race; (**E, F)** Age; **(G, H)** WBC count; **(I, J)** BM blasts; **(K, L)** PB blasts; *p* < 0.05 is considered as significant. *p < 0.05, **p < 0.01.

To explore the relationship of OS between ARNTL and clinicopathological features, we analyzed the ARNTL among diverse subgroups that were divided based on gender (Figures 4A,B), race (Figures 4C,D), age (Figures 4E,F), WBC count (Figures 4G,H), BM blast (Figures 4I,J), and PB blast (Figures 4K,L).

We found that ARNTL was identified as a risk factor in weight race human (*p* = 0.017). When the WBC count was <20, ARNTL overexpression predicted a poor prognosis (*p* = 0.002).

### The correlation of ARNTL and immune cell infiltration

**FIGURE 5:**
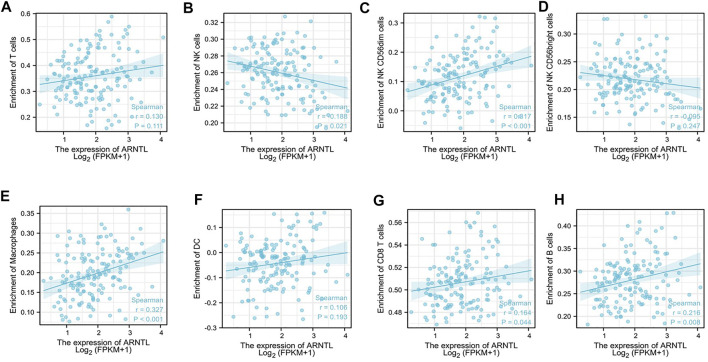
Correlation of ARNTL expression with immune infiltration level in AML. ARNTL expression has no relation with T cells **(A)**, NK cd56bright **(D)** and DC cells **(F)**. ARNTL expression has significant NK cells **(B)**, NK cd56dim **(C)**, Macrophage **(E)**, CD8+T cells **(G)** and B cells **(H)**. *p* < 0.05 is considered as significant.

We assessed the correlation between the ARNTL expression and 8 kinds of immune cells within the immune infiltration microenvironment in AML. The function of the immune cell infiltration has a significant relationship with the tumor microenvironment (TME). The results showed total NK cells (Figure 5B), NK cd56dim cells (Figure 5C), macrophages (Figure 5E), CD8^+^ T cells (Figure 5G), and B cells (Figure 5H) were correlated with the ARNTL expression. On the other hand, T cells (Figure 5A), NK cd56bright (Figure 5D), and DC cells (Figure 5F) were not related with the ARNTL expression.

### Targeting inhibition of ARNTL suppresses the malignant progression of AML

**FIGURE 6:**
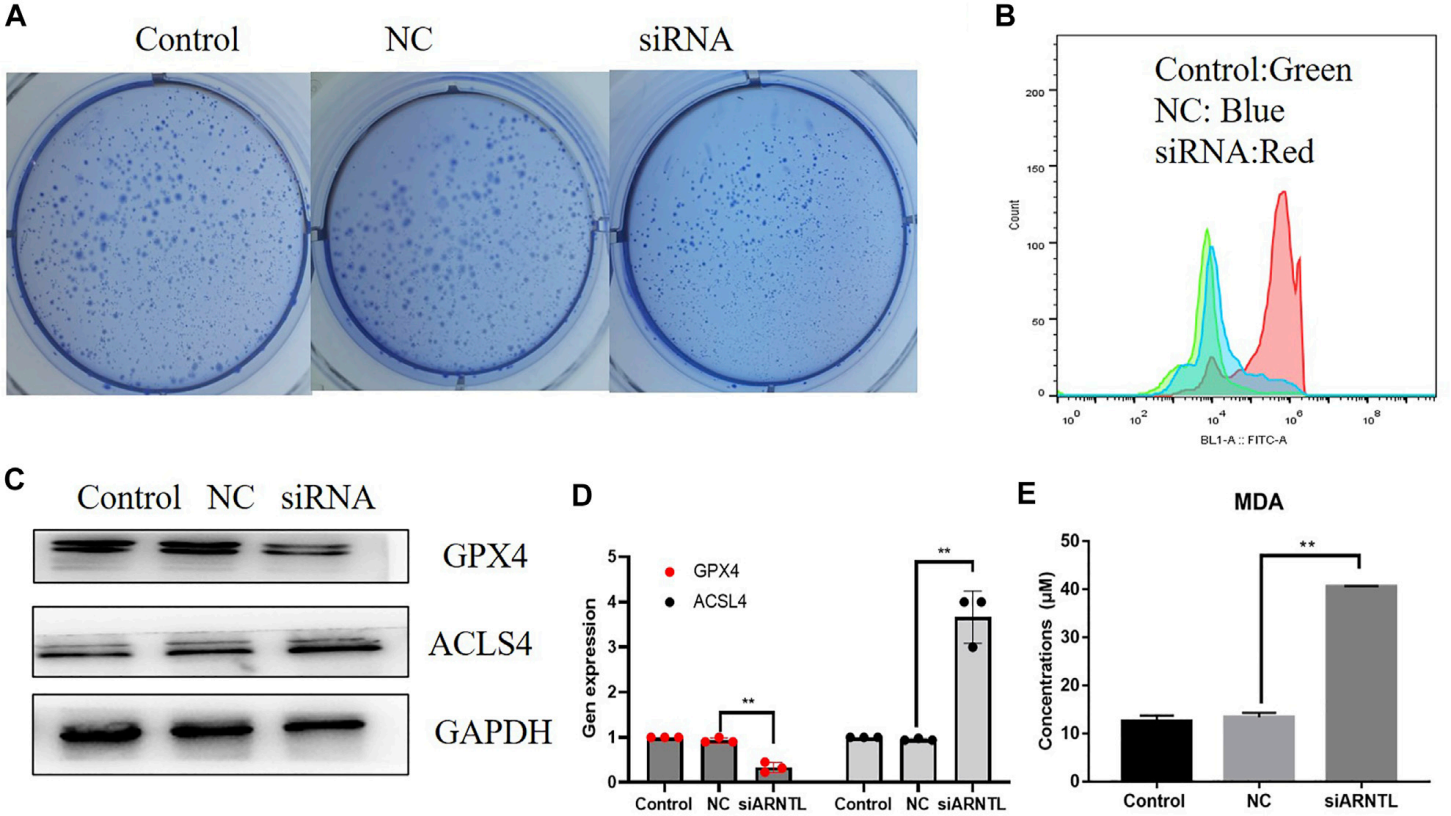
Effects of the ARNTL expression in AML cancer cell lines *in vitro*. **(A)** siARNTL inhibits colony formation in AML cells. **(B)** siARNTL promoted lipid-ROS production in AML cells. **(C)**. The effect of ferroptosis-related protein expression after the siARNTL treatment. **(D)**. The effect of ferroptosis-related gene expression after the siARNTL treatment. **(E)**. The effect of MDA production after the siARNTL treatment. *p < 0.05, **p < 0.01.

We attempted to determine whether reducing ARNTL levels through the siARNTL treatment could attenuate the AML malignant behavior. We observed the expression of ARNTL in a different type of AML cell lines from the CCLE database (Supplementary Figure S2), and we found in Molm-13 cells that ARNTL had higher expression than other cell lines, so we chose it to make further research. After ARNTL siRNA transfection, there was a substantial decrease in the clone-forming ability of Molm-13 cells relative to NC-transfected cells (Figure 6A). Therefore, these results indicate the ARNTL expression levels within malignant phenotypes in Molm-13 cells.

### ARNTL siRNA promotes lipid peroxidation levels in Molm-13 cells

Our results showed that ARNTL siRNA increased intracellular lipid-ROS. Accordingly, a substantial increase in the lipid-ROS was found after transfecting with ARNTL siRNA, indicating the increased oxidation status (Figure 6B). In addition, MDA is a lipid peroxidation marker, so the Molm-13 cell MDA levels were assayed after the ARNTL siRNA treatment. The results showed that the average level of MDA in the treated group was 3.75 ± 0.50 mmol/g, while that in the control group was 2.12 ± 0.24 mmol/g, as shown in Figure 6E. These results suggested that ARNTL siRNA induced significant lipid peroxidation in Molm-13 cells. Therefore, targeted inhibition of ARNTL in AML cells could induce ferroptosis.

### ARNTL siRNA inhibited GPX4 and promoted ACSL4 expression in Molm-13 cells

We transfected the ARNTL siRNA in Molm-13 cells, the transfection efficiency was measured by RT-qPCR, and about 50% of ARNTL was inhibited in Molm-13 cells. Then, the mRNA and protein levels of GPX4 and ACSL4 were analyzed. Our results revealed that the mRNA and protein expression of GPX4 were decreased (Figures 6C,D). In contrast, the ACSL4 expression was increased after ARNTL siRNA transfection (Figures 6C,D). These results further confirmed the inhibition of ARNTL-induced ferroptosis in AML cells.

## Discussion

AML is one of the most common lethal hematologic malignancies (Falini et al., 2021). The genetic and molecular features of AML have been extensively explored, and targeted anti-AML therapy can significantly improve the disease prognosis (Reville and Kadia, 2020). Therefore, it is necessary to diagnose, treat, and explore the pathogenic mechanism behind AML. Moreover, identifying efficient treatments for preventing AML development is required to reduce the significant mortality. Ferroptosis has recently been implicated as an iron-dependent form of programmed cell death in cancers (Liang et al., 2019). Its occurrence is due to lipid ROS accumulation resulting from the excessive ion content in cells (Ghoochani et al., 2021). Therefore, ferroptosis activators have been established as novel targets for therapeutics against advanced diseases (Chen et al., 2021). In this study, we observed a ferroptosis suppression gene ARNTL with significant high expression and correlated with a bad prognosis in AML. Moreover, targeted inhibition of ARNTL induced AML ferroptosis. Therefore, ARNTL could be an essential biomarker gene in AML.

Ferroptosis is caused by the loss of GPX4 (the enzyme for lipid repair) activity and lipid-ROS accumulation (Ding et al., 2021). Targeting ferroptosis is known to facilitate the ATPR-mediated AML differentiation through the ROS–autophagy–lysosomal pathway (Du et al., 2020). In AML, APR-246 can trigger early ferroptosis (Birsen et al., 2021), providing a novel strategy to cure AML.

ARNTL, belonging to the bHLH-PAS transcription factor family, can modulate the cellular circadian rhythm. The hypermethylation of the ARNTL promoter can be detected in blood cancers (Taniguchi et al., 2009). In our study, ARNTL mRNA levels increased markedly among the AML cases. Silencing of ARNTL could suppress the proliferation and colony-forming ability of AML cells. These findings suggest that ARNTL serves as a tumor stimulate gene in AML, which is inconsistent with its role in other malignancies. In many cancers, ARNTL acts as a tumor suppressor gene [24, 25]. Furthermore, ARNTL acts as a ferroptosis suppressor factor by repressing Egln2 transcription (Yang et al., 2019). Moreover, autophagic degradation of ARNTL promotes ferroptosis (Liu et al., 2019). We also found that the inhibition of ARNTL by siRNA promotes lipid ROS production and lipid peroxidation in AML cells. Meanwhile, the ferroptosis marker protein GPX4 was inhibited, while ACSL4 was increased by siARNTL. These results also found that ARNTL promotes ferroptosis in AML cells, consistent with other research (Zhang et al., 2021).

Glutathione peroxidase 4 (GPX4) plays a vital role in regulating ferroptosis. It also catalyzes the reduction of hydrogen peroxide, lipid hydro-peroxide, and organic hydroperoxides, thereby resisting cellular oxidative damage. In addition, GPX4, an antioxidant enzyme, functions to repair lipid peroxides and regulate cytokine signaling (Bersuker et al., 2019; Dong et al., 2021). Acyl-CoA synthetase long-chain family member 4 (ACSL4) substantially modulates lipid components and helps execute ferroptosis (Li et al., 2019). Our study found that GPX4 expression was inhibited, and ACLS4 was elevated with ARNTL inhibition. It further revealed that ARNTL acts as a ferroptosis suppressor in AML.

According to Figure 2, we observed that ARNTL expression was correlated with poor prognosis in AML. The clinical characteristics of AML patients are closely related to prognosis. The ROC curve was used to evaluate the discriminating ability of the ARNTL expression (Zhao et al., 2021). In predicting the 1-year, 2-year, and 3-year survival of AML patients in TCGA, the AUC was 0.533, 0.619, and 0.622, respectively. Therefore, the model has an excellent discrimination ability to predict survival.

The ferroptosis-related mechanism within AML is widely investigated. However, the relation of ARNTL levels with immune infiltration remains unclear. The AML immune subtypes indicated that time affected patient survival. We found macrophage, Tgd, neutrophils, cytotoxic cells, and NK CD56 bright cells have a positive correction with ARNTL. Our results revealed that the ARNTL has a tremendous immune ability to kill tumor cells.

Collectively, this work was responsible for constructing the ARNTL-based prognosis nomogram, which was accurate in discriminating and calibrating TCGA-AML patients. ARNTL could be a potential candidate for diagnosing and treating AML.

## Data Availability

The datasets presented in this study can be found in online repositories. The names of the repository/repositories and accession number(s) can be found in the article/Supplementary Material.

## Abbreviation

AML, acute myeloid leukemia; FRG, ferroptosis-related gene; TCGA, the Cancer Genome Atlas; ROC, receiver operating characteristic curve; lipid-ROS, lipid reactive oxygen species; DEGs, differentially expressed genes; K-M, Kaplan–Meier; MDA, malondialdehyde; GPX4, glutathione peroxidase 4; ACSL4, acyl-CoA synthetase long-chain family member 4; CCLE, cancer cell line encyclopedia.
