# Supplementary figures and images for: A ferroptosis-related gene signature and immune infiltration patterns predict the overall survival in acute myeloid leukemia patients
Source: Front Mol Biosci. 2022 Aug 15;9:959738. doi: 10.3389/fmolb.2022.959738 (PMC9421034; doi:10.3389/fmolb.2022.959738)

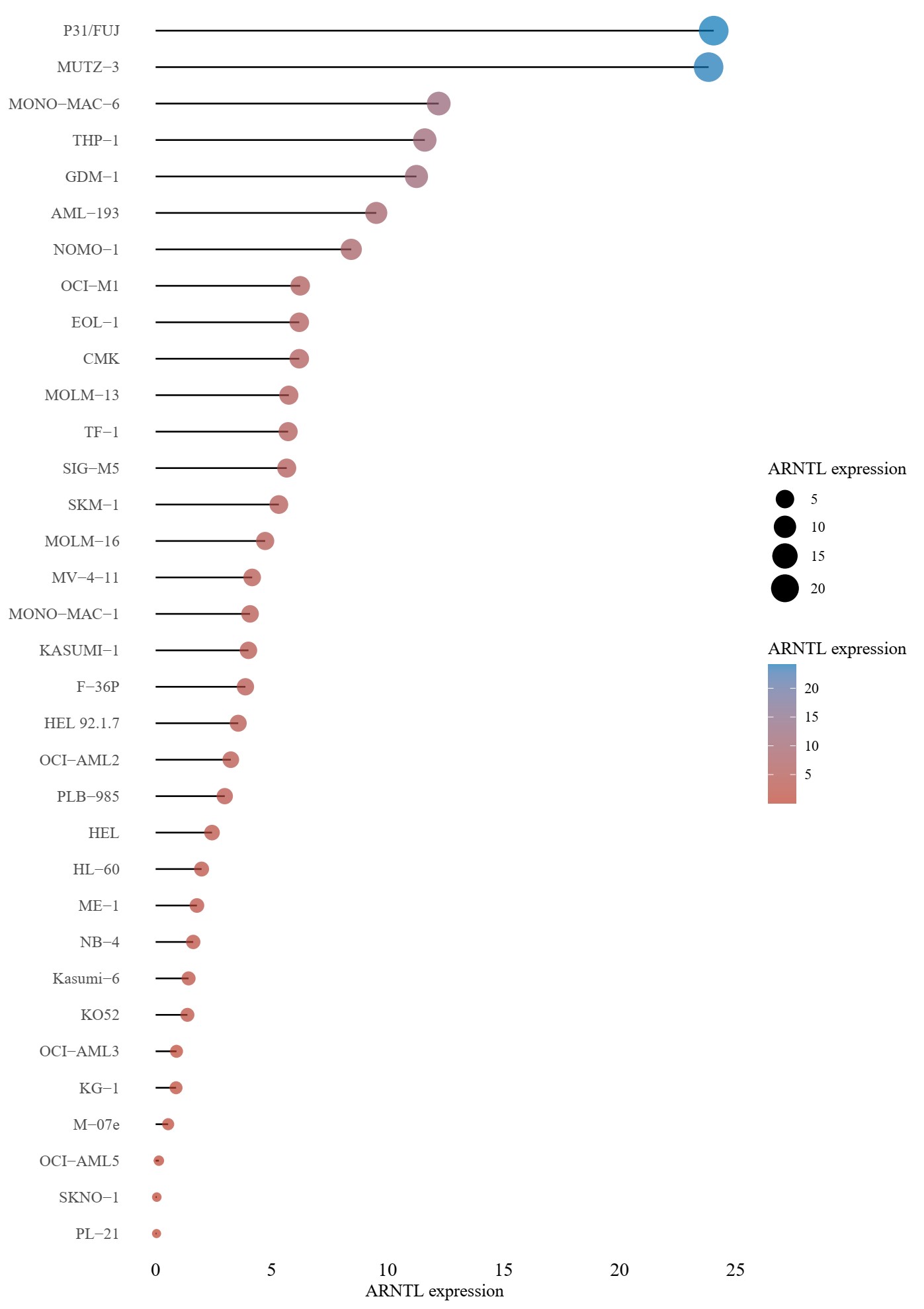

Supplement: Supplementary file 1 [file Image2.jpg]

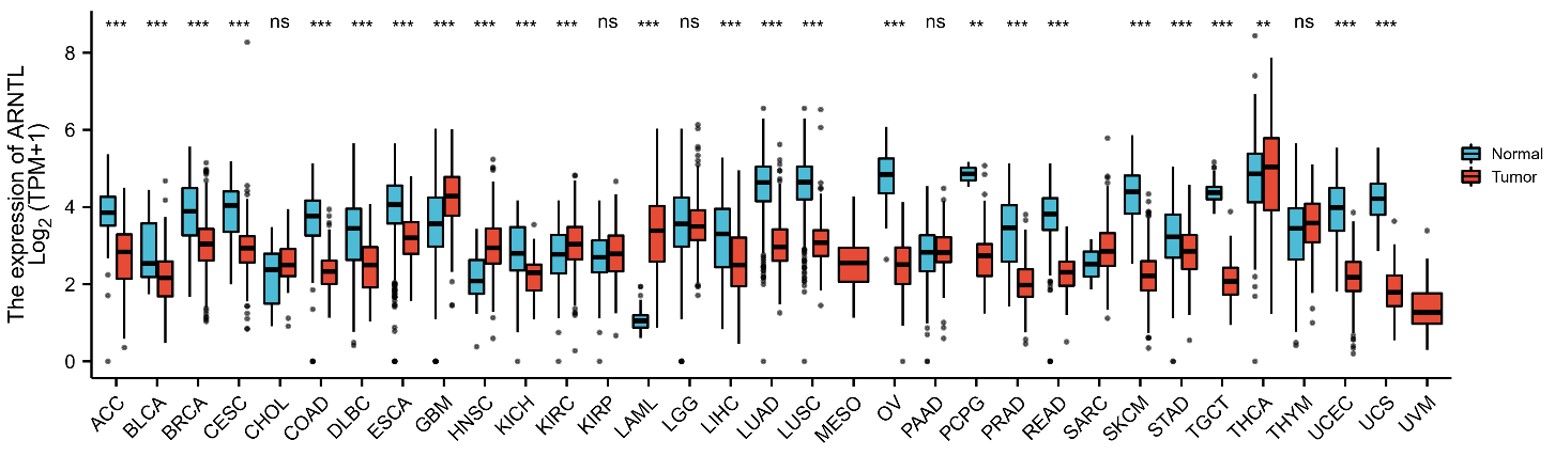

Supplement: Supplementary file 2 [file Image1.jpg]
